# Prokaryotic and Fungal Characterization of the Facilities Used to Assemble, Test, and Launch the OSIRIS-REx Spacecraft

**DOI:** 10.3389/fmicb.2020.530661

**Published:** 2020-11-05

**Authors:** Aaron B. Regberg, Christian L. Castro, Harold C. Connolly, Richard E. Davis, Jason P. Dworkin, Dante S. Lauretta, Scott R. Messenger, Hannah L. Mclain, Francis M. McCubbin, Jamie L. Moore, Kevin Righter, Sarah Stahl-Rommel, Sarah L. Castro-Wallace

**Affiliations:** ^1^Astromaterials Research and Exploration Science Division, National Aeronautics and Space Administration (NASA) Johnson Space Center, Houston TX, United States; ^2^JES Tech, Houston, TX, United States; ^3^Department of Geology, Rowan University, Glassboro, NJ, United States; ^4^Lunar and Planetary Laboratory, University of Arizona, Tucson, AZ, United States; ^5^Jacobs@NASA/Johnson Space Center, Houston, TX, United States; ^6^Astrochemistry Laboratory, Goddard Space Flight Center, Greenbelt, MD, United States; ^7^Lockheed Martin Space Systems, Littleton, CO, United States; ^8^Biomedical Research and Environmental Sciences Division, Johnson Space Center, Houston, TX, United States

**Keywords:** tag sequencing, oligotrophs, 16S, ITS, contamination, spacecraft, planetary protection

## Abstract

To characterize the ATLO (Assembly, Test, and Launch Operations) environment of the OSIRIS-REx spacecraft, we analyzed 17 aluminum witness foils and two blanks for bacterial, archaeal, fungal, and arthropod DNA. Under NASA’s Planetary Protection guidelines, OSIRIS-REx is a Category II outbound, Category V unrestricted sample return mission. As a result, it has no bioburden restrictions. However, the mission does have strict organic contamination requirements to achieve its primary objective of returning pristine carbonaceous asteroid regolith to Earth. Its target, near-Earth asteroid (101955) Bennu, is likely to contain organic compounds that are biologically available. Therefore, it is useful to understand what organisms were present during ATLO as part of the larger contamination knowledge effort—even though it is unlikely that any of the organisms will survive the multi-year deep space journey. Even though these samples of opportunity were not collected or preserved for DNA analysis, we successfully amplified bacterial and archaeal DNA (16S rRNA gene) from 16 of the 17 witness foils containing as few as 7 ± 3 cells per sample. Fungal DNA (ITS1) was detected in 12 of the 17 witness foils. Despite observing arthropods in some of the ATLO facilities, arthropod DNA (COI gene) was not detected. We observed 1,009 bacterial and archaeal sOTUs (sub-operational taxonomic units, 100% unique) and 167 fungal sOTUs across all of our samples (25–84 sOTUs per sample). The most abundant bacterial sOTU belonged to the genus *Bacillus.* This sOTU was present in blanks and may represent contamination during sample handling or storage. The sample collected from inside the fairing just prior to launch contained several unique bacterial and fungal sOTUs that describe previously uncharacterized potential for contamination during the final phase of ATLO. Additionally, fungal richness (number of sOTUs) negatively correlates with the number of carbon-bearing particles detected on samples. The total number of fungal sequences positively correlates with total amino acid concentration. These results demonstrate that it is possible to use samples of opportunity to characterize the microbiology of low-biomass environments while also revealing the limitations imposed by sample collection and preservation methods not specifically designed with biology in mind.

## Introduction

The OSIRIS-REx (Origins, Spectral Interpretation, Resource Identification, and Security–Regolith Explorer) spacecraft was designed and built to visit, study, and sample the near-Earth asteroid (101955) Bennu (Lauretta et al., 2017). The mission launched in 2016 and reached its target in December 2018. Bennu is a B-type asteroid that is spectrally similar, and therefore expected to be compositionally and mineralogically similar, to carbonaceous chondrites collected on Earth (Hamilton et al., 2019; Lauretta et al., 2019). Carbonaceous chondrites (Weisberg et al., 2006) typically contain a wide variety of organic compounds and are hypothesized to have delivered these prebiotic molecules to Earth early in its history (Lauretta et al., 2015). Some of these compounds, like amino acids (Burton et al., 2012) are easily altered by terrestrial microorganisms; others like large kerogen molecules are more recalcitrant (Kebukawa et al., 2010). Meteorites collected on Earth are often affected by microbial activity (Toporski and Steele, 2007; Steele et al., 2016; Tait et al., 2017). OSIRIS-REx will collect and return samples from Bennu using the pristine TAGSAM (Touch-and-Go Sample Acquisition Mechanism) (Bierhaus et al., 2018). The mission is required to keep these samples pristine and minimally altered during collection and return.

The OSIRIS-REx mission has a strict requirement to mitigate and document organic contamination. An extensive effort was made to characterize potential types and sources of organic and inorganic contamination during assembly, test, and launch operations by placing witness foils inside the cleanrooms where these activities occurred (Dworkin et al., 2018). NASA’s Planetary Protection guidelines designate OSIRIS-REx as a Category II outbound, Category V unrestricted sample return mission (Rummel, 2000) so there were no requirements to document biological contamination and thus a campaign of biological or DNA testing was beyond the scope of the mission. However, we had an opportunity to examine surplus witness foils originally prepared for amino acid analysis to characterize the microbiology of the assembly, test, and launch environment. This presents a rare opportunity to study a clean, but not sterilized, spacecraft over time during assembly and testing. Many cleanroom-associated microbes are capable of altering or consuming organic compounds like those we expect to return from Bennu (Balch et al., 2009; Moissl-Eichinger, 2017). Most organisms on Earth are capable of consuming amino acids which is one of the primary compound classes of interest. Even if it is unlikely that any organism will survive the harsh conditions of space for 8 years until the samples are returned to Earth, it is still useful to document microbes that the spacecraft encountered. The knowledge gained by generating a microbial inventory of organically controlled assembly, test, and launch facilities can be used to derive requirements, and develop cleaning protocols for future bioburden-controlled operations. The measurements made on the witness foil from the Atlas V fairing are especially important because every spacecraft is exposed to a similar environment before launch and the microbiological composition of this environment has never been characterized.

**FIGURE 1:**
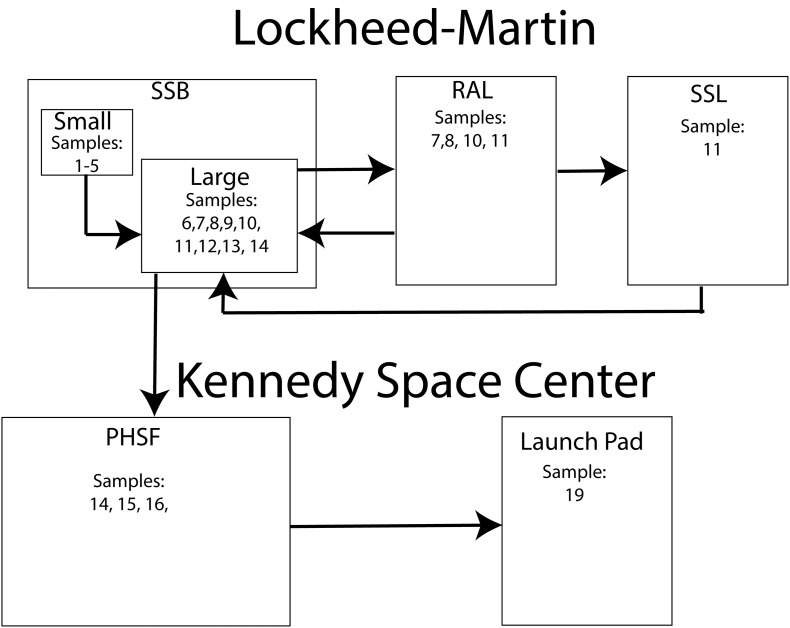
A diagram describing the ATLO locations monitored with witness foils.

The OSIRIS-REx spacecraft was constructed by Lockheed Martin Space Systems at their Waterton facility in Littleton, Colorado. The sample acquisition mechanism (TAGSAM) was assembled inside a small ISO 7 (ISO, 2015) cleanroom in the SSB (Space Science Building) before being attached to the spacecraft in a highbay ISO 7 cleanroom in the same building. The partially assembled spacecraft was then transferred to the ISO 8 RAL (Reverberation and Acoustics Lab) for vibration testing. Following vibration testing, the spacecraft was moved back to the highbay cleanroom in the SSB for further assembly. The fully assembled spacecraft was moved to the RAL, then to the ISO 8 SSL (Space Science Lab) for thermal vacuum testing and back to the highbay in the SSB. The assembled spacecraft was then packed into a N_2_ purged shipping container and flown to the Kennedy Space Center in Florida where the batteries were enabled, the sample acquisition mechanism (TAGSAM) was cleaned a final time, and the hydrazine fuel loaded onto the spacecraft. These activities occurred in the ISO 7 highbay cleanroom inside the PHSF (Payload Hazardous Servicing Facility) at the Kennedy Space Center. Finally, the spacecraft was encapsulated in an Atlas V, 4 m. Fairing, or nosecone which protects the spacecraft during launch and ascent. The encapsulated spacecraft was then driven to the launch pad under N_2_ purge where it was placed atop the Atlas V 411 launch vehicle. This entire process took 18 months (March 2015–August 2016) and each witness foil was exposed to the assembly, test, and launch environment for about 1 month or at each change in location. This process is summarized in Table 1 and Figure 1.

**TABLE 1:** Sample information.

**Sample**	**Exposure start**	**Exposure stop**	**Location**	**ATLO processes**
OR-CKP-01-1-A,0	3/11/2015	4/14/2015	LM^*a*^/Denver	SRC^*d*^ assembly. & functional. test; TAGSAM^*e*^ assembly w/clean head
OR-CKP-02-1-A,0	4/14/2015	5/11/2015	LM/Denver	TAGSAM function Development
OR-CKP-03-1-A,0	5/11/2015	6/10/2015	LM/Denver	avionics box/SRC function Post-vibrational;
OR-CKP-04-1-A,0	6/12/2015	7/14/2015	LM/Denver	SARA^*f*^ TVAC^*g*^/launch container/TAGSAM deploy. function Post-vibrational testing/OVIRS^*h*^/OTES^*i*^
OR-CKP-05-1-A,0	7/14/2015	8/19/2015	LM/Denver	TAGSAM installation, deployment.
OR-CKP-06-1-A,0	8/19/2015	9/18/2015	LM/Denver	OCAMS^*j*^ installation/SARA deployment
OR-CKP-07-1-A,0	9/18/2015	11/4/2015	LM/Denver	SARA deployment/move to RAL^*k*^
OR-CKP-08-1-A,0	11/4/2015	12/9/2015	LM/Denver	RAL vibrational testing/move to SSB^*l*^
OR-CKP-09-1-A,0	12/9/2015	1/7/2016	LM/Denver	REXIS^*m*^ OLA^*n*^ installation/spacecraft moved/shipping container
OR-CKP-10-1-A,0	1/8/2016	2/5/2016	LM/Denver	Flight. TAGSAM/spacecraft to RAL/EMI-EMC^*o*^/TVAC pre-certification.
OR-CKP-11-1-A,0	2/5/2016	3/16/2016	LM/Denver	TVAC lid opened/spacecraft to SSL/TVAC pumpdown/lid opened 3/10 move to SSB
OR-CKP-12-1-A,0	3/6/2016	4/26/2016	LM/Denver	launch container/SARA
OR-CKP-13-1-A,0	4/26/2016	4/27/2016	LM/Denver	TAGSAM flight installation
OR-CKP-14-1-A,0	4/27/2016	6/17/2016	KSC^*b*^	SARA deployment/Flight head test
OR-CKP-15-1-A,0	6/17/2016	7/14/2016	KSC	SRC battery enable/TAGSAM cleaning
OR-CKP-16-1-A,0	7/14/2016	8/26/2016	KSC	He load/prop tests/TAGSAM bottle loads/fuel sampling/cleaning & inspection/pack & shipping preparations
OR-GCKP-17-1-A1			JSC^*c*^/GSFC^*p*^	Shipping Blank
ORX Fairing Blank–FB–18			KSC	Fairing Blank
ORX Fairing CK Fck – 19	8/26/2019	9/8/2016	KSC	Fairing contamination knowledge
Swab Blank – Sb 20			JSC	
Kit Blank – Kb -21			JSC	

*^*a*^Lockheed-Martin; ^*b*^Kennedy Space Center, Florida; ^*c*^Johnson Space Center, Texas; ^*d*^Sample Return Capsule; ^*e*^Touch and Go Sample Acquisition Mechanism; ^*f*^Sample Acquisition and Return Assembly; ^*g*^Thermal Vacuum Testing; ^*h*^OSIRIS-REx Visible and Infrared Spectrometer; ^*i*^OSIRIS-REx Thermal Emission Spectrometer; ^*j*^OSIRIS-REx Camera Suite; ^*k*^Reverberation and Acoustics Lab; ^*l*^Space Sciences Building; ^*m*^Regolith X-ray Imaging Spectrometer; ^*n*^OSIRIS-REx Laser Altimeter; ^*o*^electromagnetic interference testing; ^*p*^Goddard Space Flight Center.*

In this paper, we demonstrate that contamination knowledge samples collected to monitor organic contamination can also be used to characterize microbiological changes, even when those samples are not handled aseptically or stored under conditions ideal for preserving DNA. We refer to these as, “samples of opportunity.” We successfully extracted DNA from the majority of these samples, and we describe community shifts that correspond to different portions of the assembly, test, and launch environment. The sample collected from inside the rocket fairing contained several unique organisms not present in any of the other samples. To our knowledge, this is the first time that the microbiology of the fairing environment has been described. We also describe contamination potentially introduced by utilizing samples of opportunity and discuss the limits of short-read amplicon sequencing. For example, it is not possible to definitively characterize the metabolic function of the organisms we identified in these cleanrooms due to the low amount of DNA recovered. This is the first time samples collected for organic contamination have been used for DNA sequencing. The success of this study demonstrates that organically clean contamination knowledge samples can be used for microbial monitoring even when a mission does not have microbial monitoring requirements. These types of samples could be studied from other missions with relatively little cost.

## Materials and Methods

We collected DNA from 17 witness foils and five blanks/controls. The witness foils were 1 cm^2^ squares of aluminum foil (Dworkin et al., 2018). Sixteen of the witness foils were exposed to cleanroom locations where assembly test and launch activities for OSIRIS-REx occurred. One additional foil, a 1 ft^2^ piece prepared as described above was exposed to the environment inside the Atlas V rocket fairing just prior to launch. To our knowledge this is the first microbiological measurement inside an Atlas V fairing. We extracted DNA from two control foils that were not exposed to the assembly, test, and launch environment, and three negative process controls (a blank swab, a QIAamp BiOstic Bacteremia DNA Kit with nothing added except for the included reagents, and a PCR (polymerase chain reaction) blank). The foils were organically clean and were prepared as described in Dworkin et al. (2018). Briefly, witness foils were cleaned by baking at 500°C for at least 8 h and were deployed and collected by technicians wearing nylon-free cleanroom suits with nose and mouth coverings. Technicians’ gloves were wiped with Fisher Optima 2-propanol prior to handling witness foils. We received aliquots of these witness foils in sealed glass vials (also baked at 500°C) and processed them at the Johnson Space Center microbiology lab using a previously established method. Dworkin et al. (2018), DNA was extracted by swabbing the witness foils with a polyester swab (Puritan, United States). The swab was immersed in DNA-free-water and vortexed for 5 min. A modified version of the QIAamp BiOstic Bacteremia DNA Kit (Qiagen) protocol was used to extract DNA from the swabs. We modified the kit protocol by using zircon beads to mechanically lyse the cells on the swab for 10 min prior to extracting the DNA. We also extracted DNA from the ZymoBIOMICS^TM^ Cellular Microbial Community Standard and a fungal mock community (Bakker, 2018). These mock communities (Supplementary Table S1) allowed us to describe any systematic bias in our DNA extraction and sequencing protocols. Potential contaminant sequences were included in subsequent analyses due to ambiguity in the results, which are described below (section “Bacterial and Archaeal Sequencing”).

Extracted DNA concentration was measured with a Qubit fluorimeter (Thermo Fisher Scientific) prior to amplification with 35 cycles of PCR. An Agilient 2100 Bioanalzyer was used to check the quality of the amplified product. We used the Earth Microbiome primers (Caporaso et al., 2012; Walters et al., 2015) (515F-806R and 27Fmod-519Rmod) to amplify the V4 region of the 16S rRNA gene in order to identify bacteria and archaea. We used the ITS1f-ITS2 primers to amplify the ITS1 region in order to identify fungi (Walters et al., 2015). Several insect species were observed in the cleanrooms during assembly and testing, so we also amplified the COI gene (mitochondrial cytochrome c oxidase subunit I) to identify any arthropod DNA that may have been present on the witness foils. We used the ZBJ-ArtF1c/ZBJ-ArtR2c primer pair to amplify the COI gene (Zeal et al., 2011; Madden et al., 2016). The amplified products were pooled and purified using Agencourt AMPure XP beads (Beckman Coulter). DNA was normalized and sequenced using an Illumina MiSeq with V2 chemistry for 500 cycles.

The resulting 250 base pair, paired-end reads were demultiplexed on the sequencer and processed using QIIME2 (Caporaso et al., 2010) and the Deblur pipeline (Amir et al., 2017) with a minimal read length of 120 bp and suggested parameters for quality filtering and sOTU (sub-operational taxonomic unit, 100% unique) clustering. sOTUs were identified using the SILVA v132 database and or by using BLASTn (Camacho et al., 2009) to align to the NCBI database. ITS sequences were processed using QIIME2 (Caporaso et al., 2010) and a 120 bp minimal read length in the DADA2 pipeline (Callahan et al., 2016) for quality filtering and sOTU clustering. DADA2 is preferable to Deblur for ITS sequences because it does not require uniform length reads. The length of the ITS region can vary significantly (250–400 bp) from species to species (Lindahl et al., 2013; Nilsson et al., 2019). Therefore, it is preferable to use a pipeline that can accommodate this variation.

In order to quantify the detection limits of our sequencing run, we also extracted and amplified DNA from 12 positive control samples containing 34, 340, 3,400, or 34,000 *Bacillus subtilis* cells. DNA was extracted from these cells using the Bacteremia kit described above and amplified using the 515F-806R and 27Fmod-519Rmod primer pair.

**FIGURE 2:**
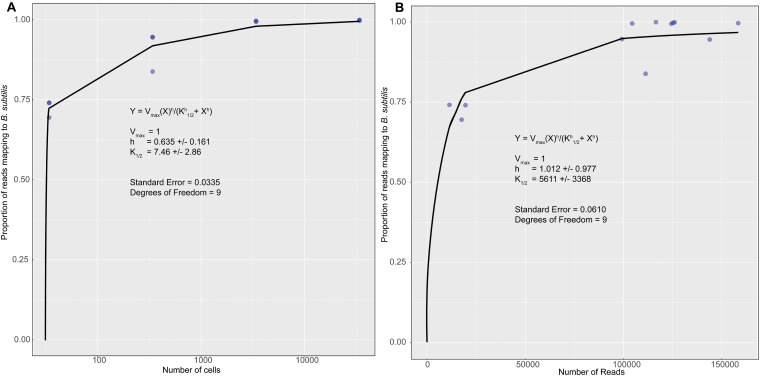
A plot of the fraction of reads for each standard that mapped to the known organism *Bacillus subtilis* vs. the number of cells in each samples **(A)** and the fraction of reads for each standard that mapped to the known organism *Bacillus subtilis* vs. the number of reads or sequences collected for each sample **(B)**. The data was fit using an allosteric sigmoidal equation. The detection limit for this sequencing run was 7 ± 3 cells and 5,611 ± 3,369 reads.

The KatharoSeq method (Minich et al., 2018) uses standards of a known organism to quantify the detection limits for a sequencing run. After sequencing, the standards and identifying sOTUs two plots are generated. Figure 2A relates the number of cells in each standard sample to the fraction of the total number of sequences assigned to the standard organism (*Bacillus subtilis*). Figure 2B relates the total number of sequences in each sample to the fraction of the total number of sequences assigned to the standard organism. Both plots are fit using an allosteric sigmoidal fitting Eq. (1) of the type

(1) $$Y=V_{m\,a\,x}^{*}\,\frac{X^{h}}{\left(K_{\text{1}\,/\,\text{2}}^{h}+X^{h}\right)}$$

**FIGURE 3:**
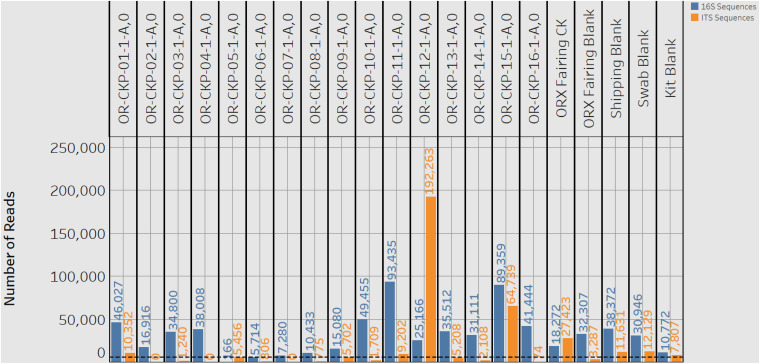
A plot showing the number of 16S rRNA gene (blue bars) and ITS (orange bars) reads or sequences for each sample. A black dashed line marks the detection limit for 16S rRNA gene sequences of 5,611 sequences. One 16S rRNA gene sample (CKP-05) and nine ITS samples fell below this limit of detection.

This equation is adopted from the field of enzyme kinetics (e.g., Childs and Bardsley, 1975). For our data K_1__/__2_ represents the detection limit. This corresponds to the point at which < 50% of the sequences are identified as *B. subtilis*. This value can be used to determine a detection limit in terms of the minimum number of cells in Figure 2A and the number of sequences or reads in Figure 2B. *V*_*max*_is a fitting parameter that represents the theoretical maximum fraction of sequences that can be identified as B. *subtilis.* In the original paper (Minich et al., 2018), this value was allowed to vary freely. However, we were unable to accurately fit our data in Figure 2B unless we forced to be one. This is a reasonable value because it is impossible for more than 100% of the reads to be identified as B. *subtilis*. For allosteric enzymes, h is a fitting parameter called the Hill coefficient. Values larger than 1 reflect stronger interactions between enzyme subunits. For our data, h is purely a fitting parameter and was allowed to vary freely. Fitting was performed in the statistical software R (version 3.6.2) using the MINPACK package to implement a Levenber-Marquardt non-linear least-squares algorithm. Fitting the data in Figure 2A gave a K_1__/__2_ value of 7 ± 3 cells as our detection limit. This corresponds to K_1__/__2_ value of 5,611 ± 3,369 reads in Figure 2B. The large standard deviation is likely reflective of error introduced prior to PCR amplification. Small amounts of contamination introduced prior to this step would have been amplified along with the target DNA and would result in large variability in the number of reads per sample. Using this information, we can assert that the swab from OR-CKP-05-1-A,0 (Figure 3) was below our limit of detection for bacterial and archaeal DNA. This sample had fewer than 5,611 reads and therefore presumably had fewer than seven cells. We did not analyze the 16S rRNA gene sequences from this sample any further.

Bacterial and archaeal communities were described using the ecological concepts of alpha and beta diversity. Analyses were chosen based on previous studies of cleanrooms (e.g., Koskinen et al., 2017; Lax et al., 2017; Minich et al., 2018) so that we could easily compare results. Alpha diversity is a measure of the number of different organisms present in a sample as well as relative abundance or “evenness” of these organisms. We chose to represent alpha diversity using the Shannon index (Shannon and Weaver, 1949). The Shannon index is an alpha diversity statistic that summarizes the number of sOTUs present in each sample and the relative abundance with which they occur. Larger numbers indicate that a sample is more diverse. Beta diversity statistics are often used to distinguish samples with many common sOTUs. Beta diversity compares the presence, absence and relative abundance of sOTUs between samples. The weighted UNIFRAC metric (Lozupone and Knight, 2005) calculates the distance between samples as a function of sOTU: presence/absence, relative abundance, and phylogeny. These distances are then analyzed using PCOA (principle coordinate analysis), a method used to explain the variance in multi-variate data sets. Samples that have a similar composition cluster together on a PCOA plot. Samples with a different composition appear as outliers. Diversity indices and PCOA values were calculated using QIIME2 (Caporaso et al., 2010).

$$Y=V_{m\,a\,x}^{*}\,\frac{X^{h}}{\left(K_{\text{1}\,/\,\text{2}}^{h}+X^{h}\right)}\,V_{m\,a\,x}\,V_{m\,a\,x}$$

We investigated correlations between sequencing results, previously published amino acid concentrations, and microscopic observations of carbon-bearing particles (Dworkin et al., 2018) using a linear regression as implemented in the Tableau software package.

## Results

### Bacterial and Archaeal DNA Sequencing

**FIGURE 4:**
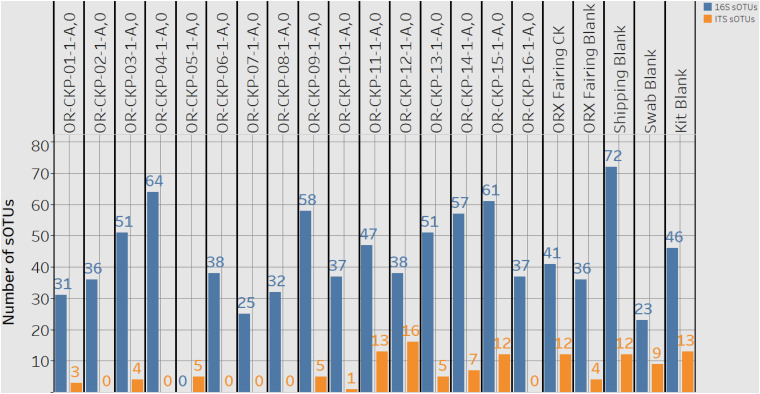
A plot detailing the number of 16S rRNA gene or bacterial sOTUs (blue bars) and ITS or fungal sOTUs identified in each sample. 16S rRNA gene samples with < 5,611 16S rRNA gene sequences are listed as having zero sOTUs. ITS samples with zero sequences also have zero sOTUs.

Extracted DNA ranged from 0.13 and 1.11 ng/μl of in our samples. After cleanup and amplification we did not observe any evidence of contamination. We observed 1,009 sOTUs across all of our samples. Most of these sOTUs were only identified in a few samples. Individual samples contained 23–72 sOTUs (Figure 4). The sample with the highest number of sOTUs (72) was the shipping blank that we used to document contamination from the sample preparation and analysis labs at Johnson Space Center and Goddard Space Flight Center. This result implies that a large portion of the bacterial diversity may be attributable to contamination from sample handling for amino acid measurements. Sample OR-CKP-04-1-A,0-CKP had the second highest number of sOTUs (64). This sample was collected while the TAGSAM was vacuum tested. The number of sOTUs does not appear to systematically change with time. The Zymo mock community was sequenced as a positive control and we were able to detect all the included bacterial species at relative abundances < 10% different than the expected abundance.

**FIGURE 5:**
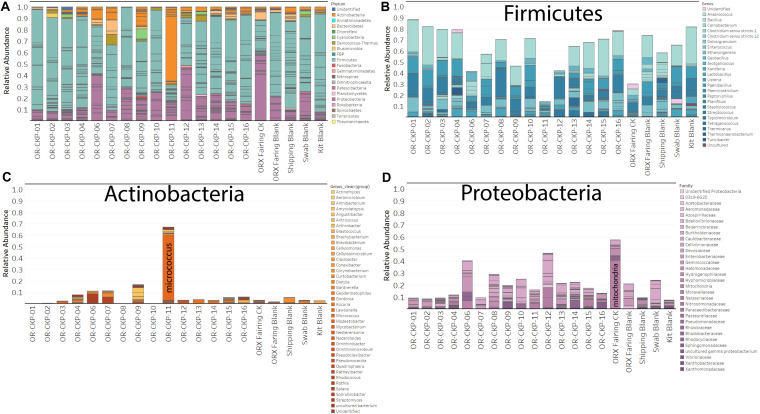
Bar charts detailing bacterial and archaeal sOTUs identified in each sample. Bars are colored by phyla in **(A)**. Abundant phyla are presented with genus level identifications **(B,C)** or family level identifications **(D)**. The most abundant phylum in most samples is Firmicutes (Blue bars; **A,B**).

**FIGURE 6:**
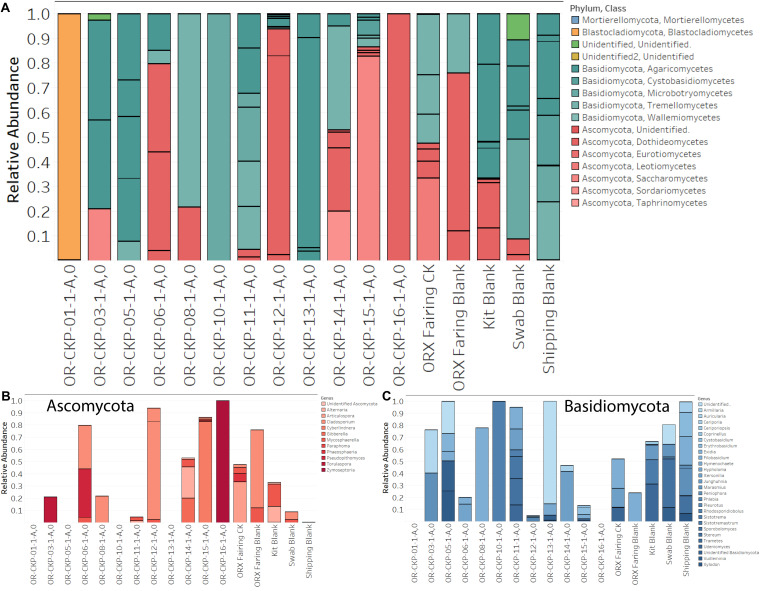
Fungal sOTUs identified in each sample **(A)**. sOTUs are colored by pyhlum and class. Most samples are dominated by sequences belonging to *Basidomycota* (blue bars; **A,C**). Samples 6, 12, 15, and 16 are dominated by *Ascomycota* (red bars; **A,B**). Abundant phyla are plotted at the genus level in **(B,C)**.

**FIGURE 7:**
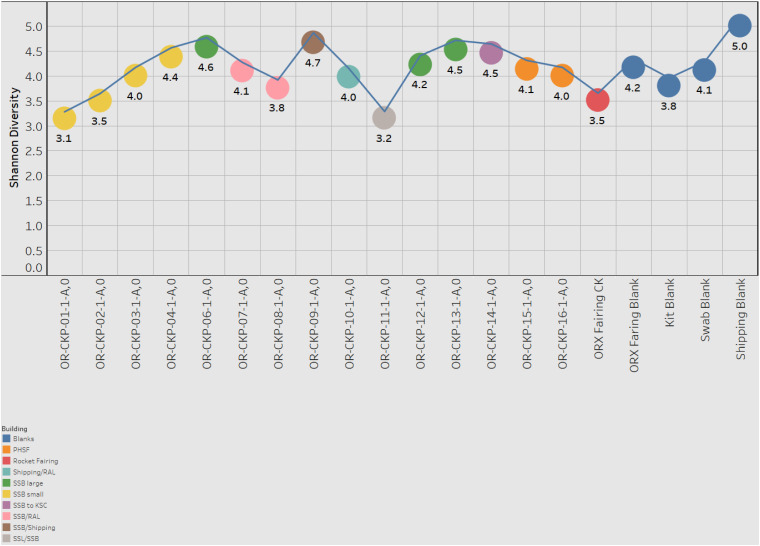
Shannon index for each sample. Samples are colored according to the building in which the witness foil was exposed.

Most of the samples are composed of a few dominant sOTUs (Figures 5A,B). These sOTUs map to human associated genera like *Bacillus, Listeria, Enterococcus*, and *Staphylococcus.* Because we were able to detect all the species included in the Zymo mock community at relative abundances < 10% different than those expected we conclude that our DNA amplification and sequencing methods did not impart any undue biases and that it is appropriate to apply alpha and beta diversity parameters. Shannon indices range from 3.2 for OR-CKP-11-1-A,0, which is dominated by *Micrococcus* sp., to 4.7 for sample OR-CKP-09-1-A, which has a more even distribution of sOTUs. The shipping blank, OR-CKP-17-1-A,0 had the highest Shannon index (5.0), which again indicates that sample handling may have artificially increased diversity. Overall, these are low diversity scores and a narrow range indicating that the samples have low diversity (Figure 6). For comparison, samples collected from the Jet Propulsion Laboratory’s Spacecraft Assembly Facility have Shannon indices as high as 6.6 (Mahnert et al., 2015) which represents a sixfold increase in diversity from a score of 4.7 because the Shannon index is on a natural log scale. The PCOA plot of the weighted UNIFRAC (Figure 7) metric contained one cluster and two outliers. The cluster contained all the blanks and control samples as well as most of the contamination knowledge samples. The two outliers were OR-CKP-11-1-A,0, exposed during thermal vacuum testing and the fairing contamination knowledge sample.

### Fungal and Arthropod Sequencing Results

We observed 87 fungal sOTUs across all our samples. As with the 16S rRNA gene sequencing most of these sOTUs only occurred in a few samples. We observed 1–16 sOTUs per sample (Figure 4). We sequenced the ZymoBIOMICS Microbial Community Standard, which contained two fungal isolates, and a mock community from the USDA (Bakker, 2018) containing 19 isolates as positive controls. We were able to detect both fungal isolates in the ZymoBIOMICS Microbial Community Standard, but the relative abundance of *Cryptococcus neoformans* was only 4.4%. The theoretical abundance in this mock community is 26% implying we are under-sampling this organism. We only detected six of the 19 isolates in the USDA mock community. These six isolates belonged to three different divisions. We did not detect organisms belonging to the *Chytridiomycota* or *Glomermomycota* divisions even though they were present in the USDA mock community.

**FIGURE 8:**
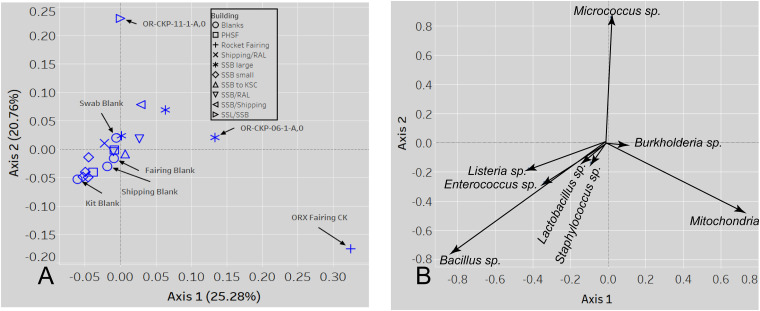
A PCOA plot of the weighted UNIFRAC beta diversity metric **(A)** shows that samples 11 and the fairing CK sample are outliers. Samples are colored by location A biplot **(B)** shows which sOTUs/organisms affect the distribution of the data. The length of the arrow is proportional to the strength of the effect.

Sample 12 had the largest number of fungal OTUs (16) (Figure 4) and the largest number of ITS sequences (Figure 4) (192,263). This sample was collected in the SSB while the launch container was being installed. In general, the samples collected after thermal vacuum testing (OR-CKP-11-1-A,0) appear to have more fungal OTUs than those collected prior to this procedure (Figure 4). Sequences belonging to the genus’s *Filobasidium* and *Cladosporium* are the most commonly occurring sequences in our samples, but they each only occur in five of the seventeen samples (Figure 8).

We were unable to detect arthropod DNA in any of our samples except for the positive controls.

## Discussion

### Bacterial and Archaeal Sequencing

We observed low numbers of sOTUs per sample (23–72) even for a cleanroom environment. For example, samples collected from the Spacecraft Assembly Facility at the Jet Proposal Laboratory contained about 100 sOTUs (Minich et al., 2018).

In general our results from sequencing the 16S rRNA gene are consistent with those reported in Dworkin et al. (2018). In that paper, six bacterial species and one archaea were detected using 16S rRNA gene sequencing. We detected the same archaeal species (*Natronococcus amylolyticus)* in our samples and two of the same bacterial species (*L. fermentum* and *Sphingobium* sp.). We detected other members of the *Pseudomonas* genus and species from the same family as *Brevibacterium* and *Reyranella*. Dworkin et al. (2018) also reported detecting *Eubacterium* sp. We did not identify any close relatives of this organism but did detect members of the same taxonomic order. The minor differences in what we were able to detect are likely due to the fact that we analyzed our samples several years after the sample reported in Dworkin et al. (2018). Since the samples were stored at room temperature, DNA from low abundance species may have degraded during that time.

The most abundant sOTU in the entire dataset was present in almost every sample, and maps to the genus *Bacillus*. The presence of this sOTU in the shipping blank suggests that it represents contamination from sample handling and storage. However, the sOTU for *Bacillus* as well as other abundant sOTUs for *Listeria, Staphyloccocu*s, and *Enterococcus* are not capable of distinguishing individual species from these genera. We used BLASTn to compare the 250 base pair sequence of the *Bacillus* sOTU to the NCBI database. This sOTU was a 100% match to six different *Bacillus* species. *Listeria, Enterococcus*, and *Staphylococcus* sOTUs matched five, four, and nine different species, respectively (Table 2). These ubiquitous sOTUs could contain multiple species that are not contaminants. We do not believe these sequences represent contamination from DNA extraction or PCR amplification because the relative abundance of sequences in the Zymo mock community matched the expected values ± 2%, and because we did not observe any evidence of contamination in the bioanalyzer data collected post-PCR. The sOTUs present in most of our samples were not present in samples from a different study sequenced during the same run, which implies that cross contamination between samples during sequencing did not occur. The low concentration of extractable DNA prevented us from reamplifying and resequencing a different region of the 16S rRNA gene to better resolve these sOTUs. This is a limitation imposed by working with samples of opportunity that were not originally collected for microbiological analysis.

**TABLE 2:** sOTUs occurring in every sample and organisms with identical sequences in the NCBI database.

**sOTU**	**Sequence**	**Organism**	**Blast Name**	**Score^*a*^**	**Number of Hits**
82595768ab5e2e41 50162ed4f0d9885a	TACGTAGGTGGCAAGCGTTGTCCGGAATTATTGGGCGTAAAGGGCTCGCAGGCGGTTCCTTAAGTCTGATGTGAAAGCCCCCGGCTCAA CCGGGGAGGGTCATTGGAAACTGGGGAACTTGAGTGCAGAAGAGGAGAGTGGAATTCCACGTGTAGCGGTGAAATGCGTAGAGATGTG GAGGAACACCAGTGGCGAAGGCGACTCTCTGGTCTGTAACTGACGCTGAGGAGCGAAAGCGTGGGGAGCGAAC				
		*Bacillus subtilis*	Firmicutes	452	20
		*Bacillus subtilis* subsp. spizizenii	Firmicutes	452	2
		*Bacillus* mojavensis	Firmicutes	452	15
		*Bacillus* halotolerans	Firmicutes	452	17
		*Bacillus* tequilensis	Firmicutes	452	2
		*Bacillus subtilis* subsp. spizizenii ATCC 6633	Firmicutes	452	1
		*Bacillus subtilis* subsp. stercoris	Firmicutes	452	1
		*Bacillus* sp. (in: Bacteria)	Firmicutes	452	32
		*Bacillus* safensis	Firmicutes	452	1
		*Bacillus* endophyticus	Firmicutes	452	1
		*Bacillaceae* bacterium	Firmicutes	452	2
		Uncultured bacterium	Bacteria	452	1
		Uncultured prokaryote	Unclassified	452	5

e336f37a27cbd76d b5b0040a8c078e20	TACGTAGGTGGCAAGCGTTGTCCGGATTTATTGGGCGTAAAGCGCGCGCAGGCGGTCTTTTAAGTCTGATGTGAAAGCCCCCGGCTTA ACCGGGGAGGGTCATTGGAAACTGGAAGACTGGAGTGCAGAAGAGGAGAGTGGAATTCCACGTGTAGCGGTGAAATGCGTAGATATG TGGAGGAACACCAGTGGCGAAGGCGACTCTCTGGTCTGTAACTGACGCTGAGGCGCGAAAGCGTGGGGAGCAAAC				
		*Listeria* monocytogenes	Firmicutes	452	36
		*Listeria* seeligeri	Firmicutes	452	4
		*Listeria* ivanovii	Firmicutes	452	2
		*Listeria* welshimeri	Firmicutes	452	1
		*Listeria* innocua	Firmicutes	452	1
		*Listeria ivanovii* subsp. londoniensis	Firmicutes	452	1
		*Listeria* sp.	Firmicutes	452	4
		Uncultured *Listeria* sp.	Firmicutes	452	51

a1a3200b76bcd600 0a0914892d370b6e	TACGTAGGTGGCAAGCGTTGTCCGGATTTATTGGGCGTAAAGCGAGCGCAGGCGGTTTCTTAAGTCTGATGTGAAAGCCCCCGGCTCAAC CGGGGAGGGTCATTGGAAACTGGGAGACTTGAGTGCAGAAGAGGAGAGTGGAATTCCATGTGTAGCGGTGAAATGCGTAGATATATGG AGGAACACCAGTGGCGAAGGCGGCTCTCTGGTCTGTAACTGACGCTGAGGCTCGAAAGCGTGGGGAGCAAAC				
		*Enterococcus* faecalis	Firmicutes	452	59
		*Enterococcus* faecium	Firmicutes	452	23
		*Enterococcus* hirae	Firmicutes	452	6
		*Enterococcus* sp.	Firmicutes	452	6
		*Enterococcus* sp. DA9	Firmicutes	452	1
		*Enterococcus* durans	Firmicutes	452	3
		*Enterococcus* faecalis EnGen0107	Firmicutes	452	1
		Uncultured bacterium	Bacteria	452	1

d23fbef2f31d48ed a40876cdbc49933a	TACGTAGGTGGCAAGCGTTATCCGGAATTATTGGGCGTAAAGCGCGCGTAGGCGGTTTTTTAAGTCTGATGTGAAAGCCCACGGCTCAAC CGTGGAGGGTCATTGGAAACTGGAAAACTTGAGTGCAGAAGAGGAAAGTGGAATTCCATGTGTAGCGGTGAAATGCGCAGAGATATGGA GGAACACCAGTGGCGAAGGCGACTTTCTGGTCTGTAACTGACGCTGATGTGCGAAAGCGTGGGGATCAAAC				
		*Staphylococcus* aureus	Firmicutes	452	35
		*Staphylococcus* hominis	Firmicutes	452	8
		Uncultured *Staphylococcus* sp.	Firmicutes	452	1
		*Staphylococcus* sp.	Firmicutes	452	9
		*Staphylococcus* epidermidis	Firmicutes	452	14
		*Staphylococcus* haemolyticus	Firmicutes	452	5
		*Staphylococcus* argenteus	Firmicutes	452	1
		*Staphylococcus* warneri	Firmicutes	452	9
		*Staphylococcus* caprae	Firmicutes	452	2
		*Staphylococcus* pasteuri	Firmicutes	452	4
		*Staphylococcus* capitis	Firmicutes	452	5
		Uncultured bacterium	Bacteria	452	4
		*Stenotrophomonas* maltophilia	G-proteobacteria	452	1
		Uncultured prokaryote	Unclassified	452	2

*^*a*^Calculated value representing the quality of the alignment see https://www.ncbi.nlm.nih.gov/books/NBK62051/ for definition.*

The largest variations in the weighted UNIFRAC metric (Figure 7) appear to correspond to times when the spacecraft was moved to a less clean environment. Sample 11 was exposed to the room containing the thermal vacuum testing apparatus. It was not possible to place a witness foil inside the test chamber. Sample 11 has a large proportion (56.2%) of *Micrococcus* reads (OTU ID: 6c891abaa8f2147383dd332e601800eb). Using BLAST (Camacho et al., 2009) this sequence matches eight different Micrococcus species: *M. aloeverae, M. yunnanensis, M. luteus, M. endophyticus, M.* sp. *KBS0714, M.* sp. *MZ2688K, M.* sp. *MZ0397G*, and *M. flavus* in the NCBI database. It is likely that this is a signal from the room where the vacuum testing occurred. *Micrococcus* species, especially *Micrococcus luteus*, are common bacteria associated with human skin and are often detected in the built environment including pharmaceutical (Utescher et al., 2007; Pacheco and Pinto, 2010) and aerospace (Puleo et al., 1973; La Duc et al., 2004; Schwendner et al., 2013; Moissl-Eichinger et al., 2015; Yang et al., 2018; Zhang et al., 2018) cleanrooms. While *Micrococcus* species are not commonly considered extremophiles *Micrococcus luteus* has been isolated from a pharmaceutical ISO 7 cleanroom maintained at 2–8°C (Sandle and Skinner, 2013). *Micrococcus* are Gram-positive cocci capable of aerobically reducing nitrate and oxidizing a variety of carbohydrates to CO_2_ and water. On human, skin these bacteria survive by consuming organic compounds in human sweat. *Micrococcus* is more prevalent on human skin during, cold winter months (Kloos and Musselwhite, 1975), which is consistent with when sample OR-CKP-11-1-A,0 was collected (February–March, 2016). We also detected a sOTU unique to this sample that belonged to the genus *Enhydrobacter* (OTU ID: 8c8598245ea67244fb156b6f31192a41) and comprised 5% of the total sequences. *Enhydrobacter aerosaccus* is the only cultivated member of this genus. This species is a Gram-negative facultative anaerobe capable of fermenting sugars and growing on a wide variety of organic compounds aerobically. It was isolated from a eutrophic lake (Staley et al., 1987). However, the genus has been detected using both culture-based and molecular methods on the International Space Station (Mora et al., 2019) and in the cleanrooms used to assemble the ExoMars 2016 mission (Koskinen et al., 2017). It is not clear what ecological niche *Enhydrobacter* is filling in the cleanroom environment, but this genus is common in aerospace cleanrooms and controlled environments like the International Space Station. We hypothesize that these organisms were shed from the skin of workers outside the test chamber and were not actively growing in the cleanroom. It is unlikely that these microbes interacted with the spacecraft given that it was isolated inside the thermal vacuum chamber, but it is encouraging that we can detect a change in the microbiome associated with moving the spacecraft to a different building in these samples of opportunity.

The samples from the small cleanroom in SSB form a very tight cluster on the PCOA plot (Figure 7A), which implies that they are highly similar. This cleanroom was used to assemble the sample return capsule and the TAGSAM portions of the spacecraft. It was one of the most stringently controlled rooms during the entire ATLO process. These four samples are dominated by the four sOTUs that occur in almost every sample, which map to the genera: *Bacillus, Listeria, Enterococcus*, and *Staphylococcus*. As discussed above, we cannot resolve these sOTUs so this may be a contamination signal from sample handling that is especially apparent in low-biomass samples. However, the sample with the lowest number of sequences (OR-CKP-06) does not cluster with these samples and, in fact, appears to have a unique bacterial signature.

Sample six has one of the highest Shannon indices of any sample analyzed (4.6) (Figure 6). The most abundant sOTU comprises 12% of the total number of sequences and is unique to sample six. The sOTU (OTU ID: f2e4355abb553aed0f53a97b59f2acf2) maps to family *Enterobacteriaceae*. Using BLASTn to compare this sequence to the NCBI database suggests that this sOTU may be from the genus *Citrobacter*, a human associated microbe commonly found in the intestinal tract that has been reported in other aerospace cleanrooms (Schwendner et al., 2013). Strains of this bacteria are capable of reducing nitrate as well as anaerobically oxidizing Fe(II) (Li et al., 2014). The other abundant, unique sOTU (OTU ID: ca3429ecc56580a91a143253d556e498) represents 7.5% of the total sequences and maps to the family *Solirubrobacter*. Despite having a smaller number of total sequences, this sample does not contain many sequences from the sOTUs that are abundant in most of the samples. It is unclear why this sample should be an outlier, as it does not correspond to any moves between buildings or extra activity during the ATLO process. The foil was exposed while the Sample Acquisition and Return Assembly was being installed and special care was taken to avoid organic contamination.

In the fairing contamination knowledge sample, 40.3% of the sequences correspond to mitochondrial (OTU ID: 12c056b5ee8cf31ff032dab1bbd2d1a4) DNA. In most microbiome samples, these sequences would be discarded as contaminants because we were specifically trying to amplify ribosomal DNA from bacteria and archaea. However, the sOTU from this sample was not present in any of our blanks or control samples indicating that it may be a legitimate signal. We used the BLASTn algorithm to compare this sequence to the NCBI database and discovered that it was a ≥ 97% match to mitochondrial DNA from the genus *Pseudoperonospora*. Specifically, the sequence matched *P. humuli* and *P. cubensis*. These eukaryotes belong to the Class *Oomycetes*. *Oomycetes*, commonly called water molds or downy mildew are fungus-like plant pathogens. *P. humuli* and *P. cubensis* are obligate parasites that infect members of the gourd family including squash, pumpkin, melon, and cucumber (Choi et al., 2005), which are grown in the areas surrounding the launch site in Florida. We also identified sequences from the genus *Pedobacter* (OTU ID: 5984c840c5cdeabb6540d4863e81270a) and *Halomonas* (OTU ID: cb49d9d1556e1d334127cfa72e508340) that were completely unique to the fairing and comprised > 5% of the total number of sequences for that sample. *Pedobacter* is a ubiquitous genus of soil bacteria that has been isolated from all over the world. These microbes may be especially relevant because some members of this genus are capable of crude oil degradation (Chang et al., 2017). Microbes that can degrade crude oil may be more able to degrade complex organic molecules like cyclo-alkanes in carbonaceous chondrites. Any of these organisms are likely capable of consuming amino acids and simple sugars like ribose (Furukawa et al., 2019). The *Halomonas* genus consists of bacteria from high salt environments like the estuaries and marine environments surrounding the launch site (e.g., Gunde-Cimerman et al., 2018). Detecting unique sOTUs in the fairing sample that are likely to have come directly from the environment surrounding the launch site implies that conditions inside the fairing prior to launch were not completely pristine. We hypothesize that (1) these microbes are indicative of contamination from the surrounding environment, and (2) they were not actively growing inside the fairing.

Although the fairing was cleaned to UV clean-highly sensitive levels prior to use and kept under a positive-pressure nitrogen purge encapsulation, then under flowing filtered air with gowned technicians working generally downstream of the witnesses (Dworkin et al., 2018), it seems likely that some amount of exchange with the surrounding environment occurred. Finding evidence for a unique microbiome inside the fairing is significant because this microbiome has never been characterized despite being an environment that every robotic spacecraft launched from Kennedy Space Center experiences. Missions with strict planetary protection requirements may need to monitor this environment to ensure that these requirements are met going forward.

### Fungal Sequencing

Previous sequencing of a contamination knowledge witness plate identified six fungal species (Dworkin et al., 2018). We detected organisms from the genus *Fusarium* but not *F. cerealis* specifically. We did not detect any *Penicillium*, but we did detect other organisms from the same class and members of the same order as *C. intermedia* and *Phoma* sp. We were unable to detect any organism closely related to *Malassezia restricta*. These differences are likely due to sample degradation caused by non-ideal storage conditions. It appears that this effect was more pronounced for ITS amplicons than it was for 16S rRNA gene sequences.

Our failure to detect 13 of the 19 species present in the USDA mock community (Bakker, 2018), indicates that there was significant bias imparted by our workflow. It is possible that the primers we chose are inappropriate. Although we chose primers validated by the Earth Microbiome Project (Walters et al., 2015) and used to characterize the USDA mock community (Bakker, 2018), several researchers have suggested that there are better suited primer sets available for ITS sequencing that produce less biased results (Bokulich and Mills, 2013; Lindahl et al., 2013; Tedersoo and Lindahl, 2016; Usyk et al., 2017). Unfortunately, we do not have enough remaining material to re-amplify our DNA with different primers. It is also possible that aggressive bead-beating to attack bacterial spores damaged the fungal DNA, or that we are eliminating valid sequences during our analysis process. Bakker (2018) reported such problems in his own analysis although to a much lesser extent. He was able to detect 17 of the 19 species in the mock community. In order to determine if our bioinformatics pipeline was responsible for our results, we processed the data using several different tools. Amplifying the ITS1 region inevitably captures some of the proceeding SSU (small sub unit) and following 5.8S genes (Nilsson et al., 2019). Some researchers suggest that better results are obtained by removing this overhang and only analyzing the ITS region (e.g., Lindahl et al., 2013; Nilsson et al., 2019). We attempted this method using ITSxpress in QIIME2 (Rivers et al., 2018), and independently using ITSx (Bengtsson-Palme et al., 2013) as implemented in the PIPITS pipeline (Gweon et al., 2015) with little success. All of these tools produced results that had even fewer species present in the mock community sample than directly processing the sequences using the DADA2 pipeline (Callahan et al., 2016) without extracting the ITS region. Due to the bias in our dataset, we chose not to employ standard ecological analysis methods like alpha and beta diversity. These calculations rely on a fully accurate measurement of the type and abundance of organisms present. The results obtained by sequencing the mock communities suggest that we did not obtain this result.

Even though our fungal sequencing results are biased, it is still useful to examine them especially when we have evidence from the bacterial and archaeal sequencing or from previous chemical measurements that changes in the assembly, test, and launch environment were occurring. For example, the fairing sample contained DNA from several unique bacterial species that are likely from the surrounding environment. We also identified several unique fungal species in this sample. The most abundant sequences (33% of the total) in the sample map to *Articulospora proliferata*. *A. proliferata* is a hyphomycete or mold. It has been isolated from leaf litter in freshwater environments. Sequences mapping to *Udeniomyces pyricola* were also abundant (11.7% of the total sequences) and unique to the fairing sample. *U. pyricola* is also a leaf degrader, but it is classified as a yeast due to its ability to reproduce by budding. *U. pyricola* has been isolated from glacial ice in Patagonia (de Garcia et al., 2012) and so is reported to be cold-tolerant. *Itersonilia pannonica* was also unique and represented 15.8% of the total sequences. The *Udeniomyces and Itersonilia* genuses are capable of producing teliospores, a unique type of spore that contains two cells per spore (Weiss et al., 2014). Sequences mapping to *Phaeosphaeria caricicola* (another leaf degrading fungi) represented 6.8% of the total sequences and were also unique to the fairing. Again, we hypothesize that these organisms are indicative of contamination from the launch environment in Florida and not indicative of fungal growth inside the fairing.

Sample 11 only contained one sOTU that was unique to the sample. The sOTU maps to the genus *Sistotremastrum* and represents 13.3% of the sequences. This is a genus of saprophytic fungi associated with white rot and lignin degradation (Nguyen et al., 2016). Samples 1, 12, and 16 appear to be dominated by individual sOTUs. Sample one is dominated by an unidentified *Blastocladiales*. The closest relative is *Brunneoporus minutus* (97% similar), which is also known as *Antrodia minuta*, a lignin degrading fungi responsible for cellulose degradation or brown rot (Spirin, 2007). Sample 12 is dominated by *Cladosporium deliculatulum* (72.5%), a more common type of indoor fungi (Yang et al., 2016). This species was also found in sample six (36%), the fairing sample (2.5%), the swab blank (6.3%), and the fairing blank (64%), which suggests that it may be contamination due to sample handling. Sample 16 is dominated by a sOTU mapping to the genus *Zymoseptoria*, which contains mostly plant pathogens (Quaedvlieg et al., 2011).

In general, our samples are dominated by the phyla *Ascomyctoa* and *Basidiomycota*. Genus *Itersonilia* found in samples 11 (17.3%), sample 12 (0.7%), sample 14 (42%), and the fairing sample (16%) belongs to the class *Tremellomycetes*. Other species from this class were also identified in sample five and sample eight (7 of 14 samples). This class is composed of wood degrading fungi, micro-fungi, gelatinous fruiting fungi, opportunistic pathogens, and parasitic fungi that live inside other fungi. These fungi are ubiquitous in the environment and have even been cultivated in samples from the Antarctic Dry Valleys (Weiss et al., 2014). Class *Agaricomycetes* is found in 8 of the 13 samples (3, 5, 6, 11, 12, 13, 14, and 15). This class includes plant pathogens and saprotrophs associated with lignin degradation or white rot (Hibbett et al., 2014). Both classes have been detected in Jet Propulsion Laboratory cleanrooms used to assemble spacecraft (La Duc et al., 2012; Checinska et al., 2015). However, their ecological function in the built environment remains uncharacterized (Samson, 2011). Perhaps the sample to sample variation represents a seasonal pattern as described by Weikl et al. (2016). Sample 12 has the highest largest number of fungal sequences and corresponds to a month with above average rainfall in the Denver area. Even in cleanrooms fungi, from the surrounding environment seems to predominate over fungi associated with human activity (Adams et al., 2013). Fungal ecology in the built environment is poorly described in the literature and further research is needed to understand how these organisms survive in cleanroom environments.

In natural environments, fungi are well adapted to utilizing recalcitrant carbon compounds like lignin. In the built environment, different fungal species have been reported growing on and degrading: wood, paper, and gypsum based plaster, polyethylene, polyvinyl chloride, and even formaldehyde based resin used in fiberglass ceiling tiles (Yang et al., 2016). Although it is unlikely that any fungal spores survived the journey to Bennu, fungi in cleanrooms on Earth are some of the best suited organisms to degrade organic compounds like kerogen (e.g., Wong et al., 2015). Kerogen along with many other organics, is expected to be a component of the returned samples (Kebukawa et al., 2010). Interestingly, we observed a weak negative correlation between the number of fungal sOTUs identified in the samples and relative abundance of carbon-bearing particles as previously identified with a scanning electron microscope on the witness plates (Dworkin et al., 2018; Supplementary Figure S1). One explanation for this observation is that fungi in the cleanroom were consuming carbon and lowering the relative abundance on witness plates. The number of bacterial sOTUs did not show this negative correlation, which is surprising considering that many members of the *Bacillus* genus are also capable of degrading recalcitrant carbon (e.g., da Cunha et al., 2006). Unfortunately, we were not able to analyze these particles to determine whether the carbon was biologically available. Additional research is required to test this hypothesis. The number of fungal ITS sequences are weakly correlated with the measured amino acid concentration (Supplementary Figure S2A). It is possible that samples with higher amino acid concentrations also had higher levels of microbial activity and thus larger amounts of detectable DNA. Amino acids can serve as a carbon source for most types of bacteria and fungi and thus prevalent organic contamination could exacerbate biological contamination. An alternate explanation is that fungal ingress into the lab was weakly correlated with lapses in cleaning or gowning procedures. Things like air leaks, missed wipe downs, and incorrectly donning and doffing cleanroom garments could have introduced amino acids and fungi. This is consistent with the observations of Adams et al. (2013) that most fungi are not endemic to cleanrooms but are introduced from the external environment. We were unable to correlate any other aspect of this dataset with amino acid concentration (Supplementary Figure S2B).

We successfully used samples of opportunity to extract and sequence bacterial, archaeal, and fungal DNA from witness foils present during assembly test and launch operations for the OSIRIS-REx spacecraft. Bacterial sequences were dominated by human associated microbes and appear to vary slightly from cleanroom to cleanroom. While it was not possible to conclusively describe the ecological niches occupied by these bacteria, it is plausible that they survive in the cleanrooms by consuming cleaning products like isopropyl alcohol (Mogul et al., 2018). Sequencing standards allowed us to quantify the minimum number of bacterial cells that we were able to detect at seven cells per sample. Fungal sequences were dominated by two fungal phyla commonly reported in cleanrooms, but these results are likely biased due to inherent limitations in DNA sequencing and data processing technologies. Further research is needed on fungal metabolism in cleanrooms. A witness foil from inside the rocket fairing contained bacterial, fungal, and other eukaryotic DNA from organisms that were not detected anywhere else during assembly or testing. This is the first time that the microbiology of a rocket fairing has been studied and demonstrates that microbial contamination of outbound spacecraft is possible even when the fairing is visibly cleaned and subsequently maintained with a positive pressure nitrogen purge.

Many of the potential contamination issues we encountered were caused by low biomass and were exacerbated by limited sample quantity and sample handling protocols that were not designed with DNA sequencing or any type of microbiological analysis in mind. Although using samples of opportunity for microbiology is possible and has generated important information about organisms present in the assembly, test, and launch environment, we recommend collecting dedicated contamination knowledge samples for DNA sequencing if microbial contamination is of interest (McCubbin et al., 2019). Dedicated samples should be frozen to preserve DNA and would allow potential contamination due to sample handling to be addressed by reanalyzing samples to amplify different genetic regions that are more discriminatory for the species we detected (e.g., *Bacillus*). Our inability to distinguish individual species of the *Bacillus, Listeria, Staphyloccocus*, and *Enterococcus* genuses demonstrates the limitations of “universal” PCR primers. The V4 region of the 16S rRNA gene amplified by the Earth Microbiome primers does not appear to be useful for distinguishing members of the genuses at the species level. If we had collected and stored samples specifically for DNA sequencing, we may have had enough material to reanalyze these samples using different primers that better discriminate these organisms, but for this study we were limited to small samples of opportunity. Collecting dedicated samples with the intent of microbiological analysis would be especially useful for missions with strict planetary protection requirements. Our results from inside the rocket fairing should also prompt more detailed study of this environment as it is unclear whether the signal that we detected represents microbial incursion from the launch environment or microbes that remain on the inside of the fairing after cleaning.

The Astromaterials Acquisition and Curation Office at Johnson Space Center is already taking precautions to limit microbial contamination in curation facilities. Routine microbial monitoring of existing curation cleanrooms is ongoing, and the results will inform new sterilization and cleaning protocols for the OSIRIS-REx lab that is currently under construction. Astromaterials curators will also collect soil from the landing sight of the Sample Return Capsule in Utah to characterize the microbes present there. Finally, it may be beneficial to try to detect DNA inside the Sample Return Capsule after the samples have been removed. The presence of identifiable DNA inside the Sample Return Capsule could indicate the microorganisms survived the trip to and from Bennu. Further research is needed to describe how bacterial and fungal growth could alter returned samples. We recommend using carbonaceous chondrites as analog materials for this work so that cleaning protocols designed to limit inorganic, organic, and biological contamination can be developed prior to the return of samples from Bennu (McCubbin et al., 2019).

## Data Availability Statement

The datasets generated for this study can be found in the SRA database https://www.ncbi.nlm.nih.gov/sra/PRJNA602955 under accession number PRJNA602955.

## Author Contributions

AR assisted with DNA extraction, processed the sequencing data, and prepared the manuscript for publication. CC, RD, SS-R, and SC-W extracted and sequenced the DNA and provided input on the manuscript. SM prepared the witness foils. HM and JD collected the witness foils, performed the amino acid analysis, and provided input on the manuscript. KR oversaw witness foil deployment, coordinated additional witness foil measurements, and provided input on the manuscript. HC, DL, and FM participated in discussions and provided substantive input on the manuscript. JM operated the facilities used to construct the spacecraft and provided input on the manuscript. All authors contributed to the article and approved the submitted version.

## Conflict of Interest

CC and SS-R were employed by the company JES Tech. RD was employed by Jacobs@NASA/Johnson Space Center. JM was employed by Lockheed Martin Space Systems. The remaining authors declare that the research was conducted in the absence of any commercial or financial relationships that could be construed as a potential conflict of interest.

## Supplementary Material

Supplementary Figure 1 The number of fungal sOTUs (orange symbols) displays a weak inverse correlation to the proportion of carbon-bearing particles on the witness foils. The number of bacterial sOTUs (blue symbols) displays a weak positive correlation to the proportion of carbon bearing particles. Symbol shape corresponds to the building in which the sample was collected.

Supplementary Figure 2 The number of fungal ITS sequences (orange symbols) is correlated with the amino acid concentration **(A)**. The number of 16S rRNA gene bacterial and archaeal sequences (blue symbols) is weakly correlated with amino acid concentration. The number of sOTUs observed does not appear to correlate to amino acid concentration **(B)**. Symbol shape corresponds to the building in which the sample was collected.
