# Supplementary figures and images for: Prokaryotic and Fungal Characterization of the Facilities Used to Assemble, Test, and Launch the OSIRIS-REx Spacecraft
Source: Front Microbiol. 2020 Nov 5;11:530661. doi: 10.3389/fmicb.2020.530661 (PMC7676328; doi:10.3389/fmicb.2020.530661)

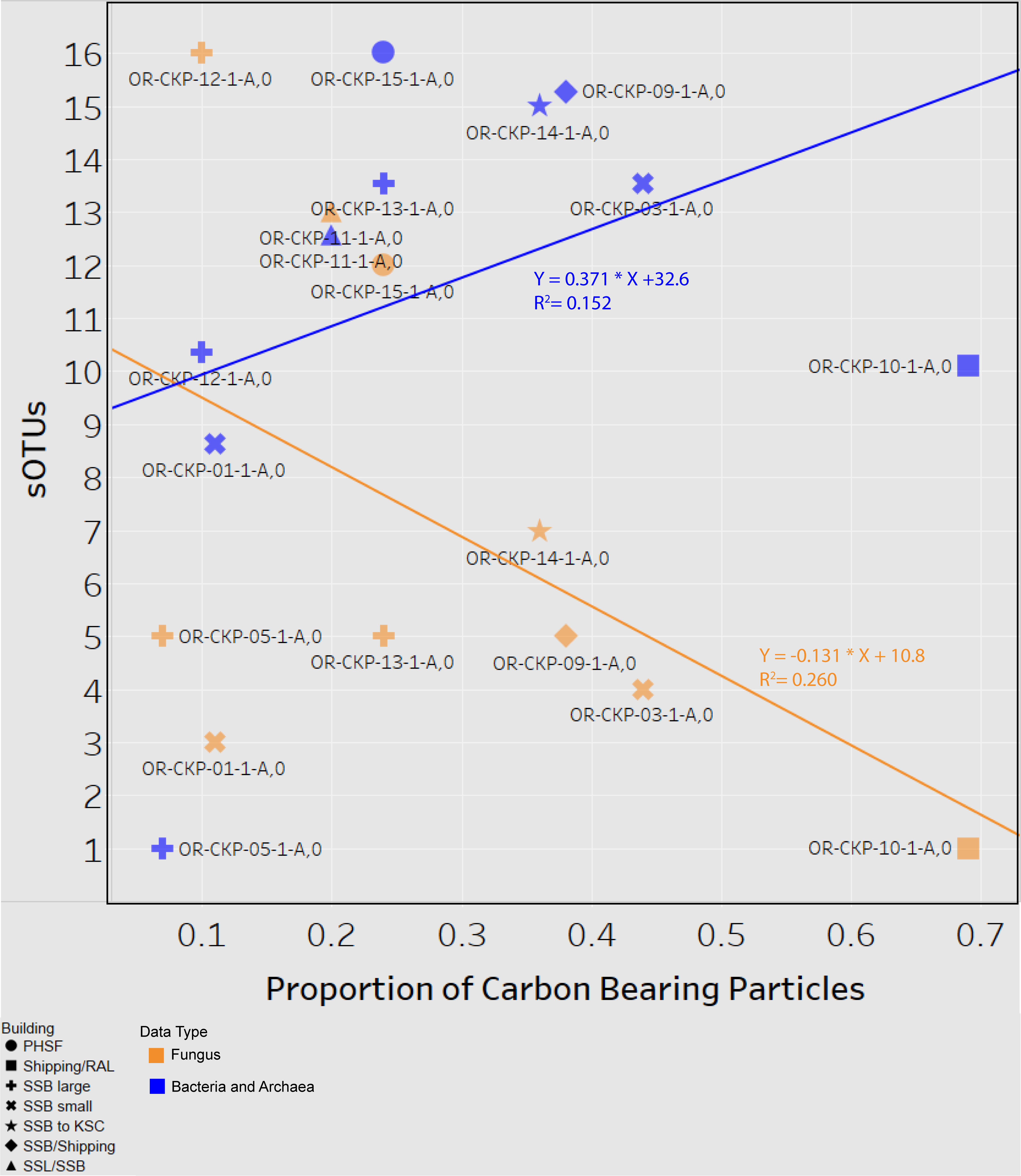

Supplement: Supplementary Figure 1 — The number of fungal sOTUs (orange symbols) displays a weak inverse correlation to the proportion of carbon-bearing particles on the witness foils. The number of bacterial sOTUs (blue symbols) displays a weak positive correlation to the proportion of carbon bearing particles. Symbol shape corresponds to the building in which the sample was collected. [file Data_Sheet_1.ZIP › Supplementary_Information/Figure_S1_C_vs_sOTUs.tif]

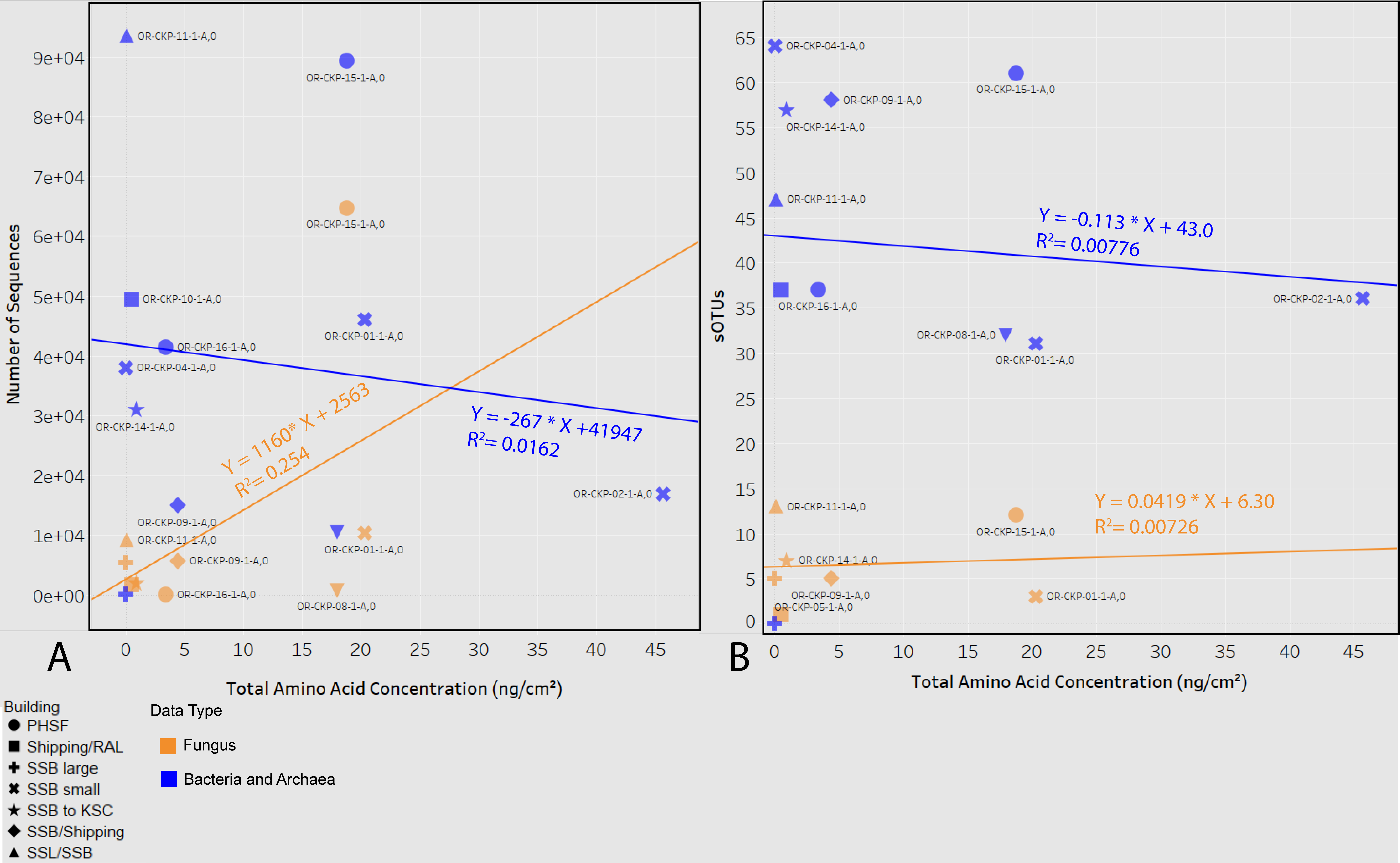

Supplement: Supplementary Figure 1 — The number of fungal sOTUs (orange symbols) displays a weak inverse correlation to the proportion of carbon-bearing particles on the witness foils. The number of bacterial sOTUs (blue symbols) displays a weak positive correlation to the proportion of carbon bearing particles. Symbol shape corresponds to the building in which the sample was collected. [file Data_Sheet_1.ZIP › Supplementary_Information/Figure_S2_Amino_acid_plots.tif]
